# Clostridium butyricum and Chitooligosaccharides in Synbiotic Combination Ameliorate Symptoms in a DSS-Induced Ulcerative Colitis Mouse Model by Modulating Gut Microbiota and Enhancing Intestinal Barrier Function

**DOI:** 10.1128/spectrum.04370-22

**Published:** 2023-03-28

**Authors:** Xingxi Huang, Jutuan Hu, Heng Zhang, Ji Li, Xi Zhu, Yangyang Liu, Yunxiang Liang, Yuxia Mei

**Affiliations:** a State Key Laboratory of Agricultural Microbiology, College of Life Science and Technology, Huazhong Agricultural University, Wuhan, People’s Republic of China; Tainan Hospital, Ministry of Health and Welfare

**Keywords:** ulcerative colitis, synbiotic, *Clostridium butyricum*, chitooligosaccharides, cytokines, gut microbiota

## Abstract

Effects of Clostridium butyricum and chitooligosaccharides (COS), singly and in synbiotic combination, were evaluated in a C57BL/6 mouse model of dextran sulfate (DSS)-induced acute ulcerative colitis (UC). Treatment with C. butyricum and/or COS ameliorated UC symptoms *in vivo*, and the strongest effects were observed for the combination in terms of reduced mortality rates and disease activity indices, increased body weight and colon length, and improved histological features. The C. butyricum and COS combination achieved the following: (i) regulated levels of inflammation-related cytokines (tumor necrosis factor alpha [TNF-α], interleukin-1β [IL-1β], IL-6, IL-10) and had a stronger anti-inflammatory effect than either component alone, based on inhibition of Toll-like receptor 4 (TLR-4)/NF-κB/MAPK signaling pathway activation; (ii) enhanced intestinal barrier function by restoring levels of tight junction proteins (occludin, claudin-1, ZO-1) and MUC2; (iii) increased abundance and diversity of beneficial bacteria (gut microbiota) and reduced levels of pathogenic bacteria; and (iv) enhanced production of short-chain fatty acids. Our findings indicate that the synbiotic C. butyricum and COS combination has strong potential as a therapeutic adjuvant for UC.

**IMPORTANCE** Ulcerative colitis (UC), an idiopathic intestinal disease characterized by continuous remission/relapse inflammatory cycles in the colonic mucosal layer, has strong adverse effects on patients’ quality of life and considerable costs for health care systems. Probiotics, prebiotics, and synbiotics are regarded as potential therapeutic agents for UC, in terms of safety and efficacy. In this study, we present detailed evaluation of effects in a DSS-induced UC mouse model of a synbiotic composed of Clostridium butyricum and COS (molecular weight [MW], 2,500 Da). We found that synergistic (synbiotic) action of the C. butyricum and COS combination is more effective than either factor alone for prevention and/or therapy of UC by regulating gut microbiota and intestinal barrier function. Our findings indicate that C. butyricum and COS in combination has strong potential for development as anti-UC therapeutic drugs or adjuvant agents in pharmaceutical, food, and livestock industries. Highlights include the following. (i) The C. butyricum and COS combination ameliorated clinical UC symptoms and improved colonic morphology. (ii) The C. butyricum and COS combination displayed strong anti-inflammatory and antioxidant effects. (iii) The C. butyricum and COS combination enhanced expression of tight junction proteins. (iv) The C. butyricum and COS combination inhibited the TRL-4/NF-κB/MAPK signaling pathway. (v) The C. butyricum and COS combination modulated gut microbiota abundance and composition.

## INTRODUCTION

Inflammatory bowel diseases (IBD), comprising mainly Crohn’s disease and ulcerative colitis (UC), are chronic, relapsing diseases that typically display increasing incidence in newly industrialized countries (1). UC, an idiopathic intestinal disease characterized by continuous remission/relapse inflammatory cycles in the colonic mucosal layer, has strong adverse effects on patients’ quality of life and considerable costs for health care systems (2). Commonly used pharmacologic agents (salicylates, immunosuppressants, corticosteroids) are intended to control symptoms, maintain nutritional balance, and promote remission in UC patients; however, they often have undesirable side effects (headache, nausea, vomiting, abdominal pain, rashes) and do not prevent relapse (3).

Imbalance in the gut microbial community (dysbiosis) has been implicated in recent decades as a factor in UC pathogenesis (4). Probiotics, which help maintain gut homeostasis, have the potential to prevent, ameliorate, or cure various intestinal diseases (5). Probiotics are defined as living microorganisms that confer some health benefit on the host. Popular, widely used examples include lactic acid bacteria, *Bifidobacterium* spp., Akkermansia muciniphila, and Clostridium butyricum (5, 6). Safety and efficiency of probiotic use for maintaining UC remission have been demonstrated in clinical trials. C. butyricum is a butyrate-producing probiotic that mainly colonizes distal small intestine and colon and has positive effects in terms of gut microbiota regulation, beneficial metabolite production, and suppression of intestinal inflammation (5, 7). Studies by several groups have shown that C. butyricum induces interleukin-10 (IL-10)-producing macrophages in inflamed mucosa, repairs structural damage of tight junction (TJ) proteins, and promotes regeneration of intestinal lymphatic vessels, thereby preventing acute colitis in experimental mouse models (8–11). In human UC patients, 4-week C. butyricum therapy during endoscopic remission reduced bowel-related symptoms and improved quality of life (12).

Prebiotics are substrates that are undigestible by the host but utilized by gut microorganisms to confer some health benefit. They comprise mainly polyols, oligosaccharides, and soluble fiber (6). Studies of human UC patients and experimental mouse models have shown that fructo-oligosaccharides, inulin, galacto-oligosaccharides, β-glucan, lactulose, resveratrol, and germinated barley extracts promote proliferation of beneficial bacteria (*Lactobacillus*, *Bifidobacteria*, *Akkermansia*) and production of short-chain fatty acids (SCFAs) (13–18).

SCFAs are the main metabolite produced by anaerobic bacteria in the colon to ferment carbohydrate-like foods. The SCFAs in the intestine mainly consist of acetic acid, propionic acid, and butyric acid, and their molar ratio in the colon is about 3:1:1. Different intestinal flora produce different SCFAs. The bacteria that synthesize acetic acid are distributed in *Actinomyces*, *Bacteroidetes*, *Firmicutes*, *Proteobacteria*, and *Verrucobacteria*, while the bacteria that synthesize propionic acid and butyric acid mostly belong to *Proteobacteria* (19–21). SCFAs are involved in host metabolism and play a role in different organs and tissues such as the gut, brain, bone, and liver (22), which enables SCFAs to not only stabilize host homeostasis and inhibit intestinal inflammation but also relieve other parenteral diseases through brain-gut axis and liver-gut axis (23, 24). Therefore, SCFAs play an important role in host health.

Synbiotics (combinations of specific strains of probiotics and selected prebiotics that function synergistically) generally display greater efficacy than either probiotics or prebiotics used alone in terms of gut health and function (2, 6). Synbiotics composed of β-glucan and complex probiotics modulated inflammatory cell infiltration and inflammatory markers (IL-6, tumor necrosis factor alpha [TNF-α]) in C57BL/6 mice and had stronger and broader inhibitory effects on colonic inflammation than β-glucan or probiotics used alone (18). In studies of a UC mouse model by O'Keefe’s group, arabinoxylan (AX) and Lactobacillus fermentum HFY06 acted synergistically to ameliorate UC symptoms, reverse histopathological changes in the colon, and suppress activation of the nuclear factor κB (NF-κB) signaling pathway and release of proinflammatory cytokines (25). Similar findings were obtained for a combination of Bifidobacterium infantis and xylooligosaccharide in a UC mouse model (26). A synbiotic composed of Lactobacillus gasseri 505 and Maclura tricuspidata leaf extract displayed antitumor effects in a mouse model of azoxymethane (AOM)/dextran sodium sulfate (DSS)-induced, colitis-associated colorectal cancer, particularly in terms of reduced colonic damage and tumor incidence (27). Results of UC treatment by synbiotics are promising, although the number of studies to date is small (2).

Chitooligosaccharides (COS), products of enzymatic or chemical degradation of chitosan or chitin, have been extensively studied in recent decades because of their numerous beneficial activities, including antitumor (28), immunomodulatory (29), antioxidant (30), antimicrobial (31), and anti-inflammatory (32). Our previous study suggested potential application of COS as anti-UC prebiotics, in view of their ameliorative effect on UC resulting from altered gut microbiota composition and restoration of intestinal barrier function (33). In early weaned pigeon squabs, a combination of COS and C. butyricum, added to the diet, enhanced growth and intestinal health (34). No studies to date have addressed efficacy of UC treatment by synbiotics composed of C. butyricum and COS or the underlying mechanisms.

Here, we present detailed evaluation of effects in a DSS-induced UC mouse model of a synbiotic composed of C. butyricum and COS (MW, 2,500 Da). Parameters such as body weight, disease activity index (DAI), colonic morphology, expression levels of inflammation-related cytokines, and proteins in relation to oxidative stress levels were quantified. Underlying mechanisms were investigated based on analyses of intestinal mucosal barrier function, inflammation-related signaling pathways, and gut microbiota composition.

## RESULTS

### C. butyricum ± COS ameliorated clinical symptoms in our mouse model of DSS-induced UC.

**FIG 1:**
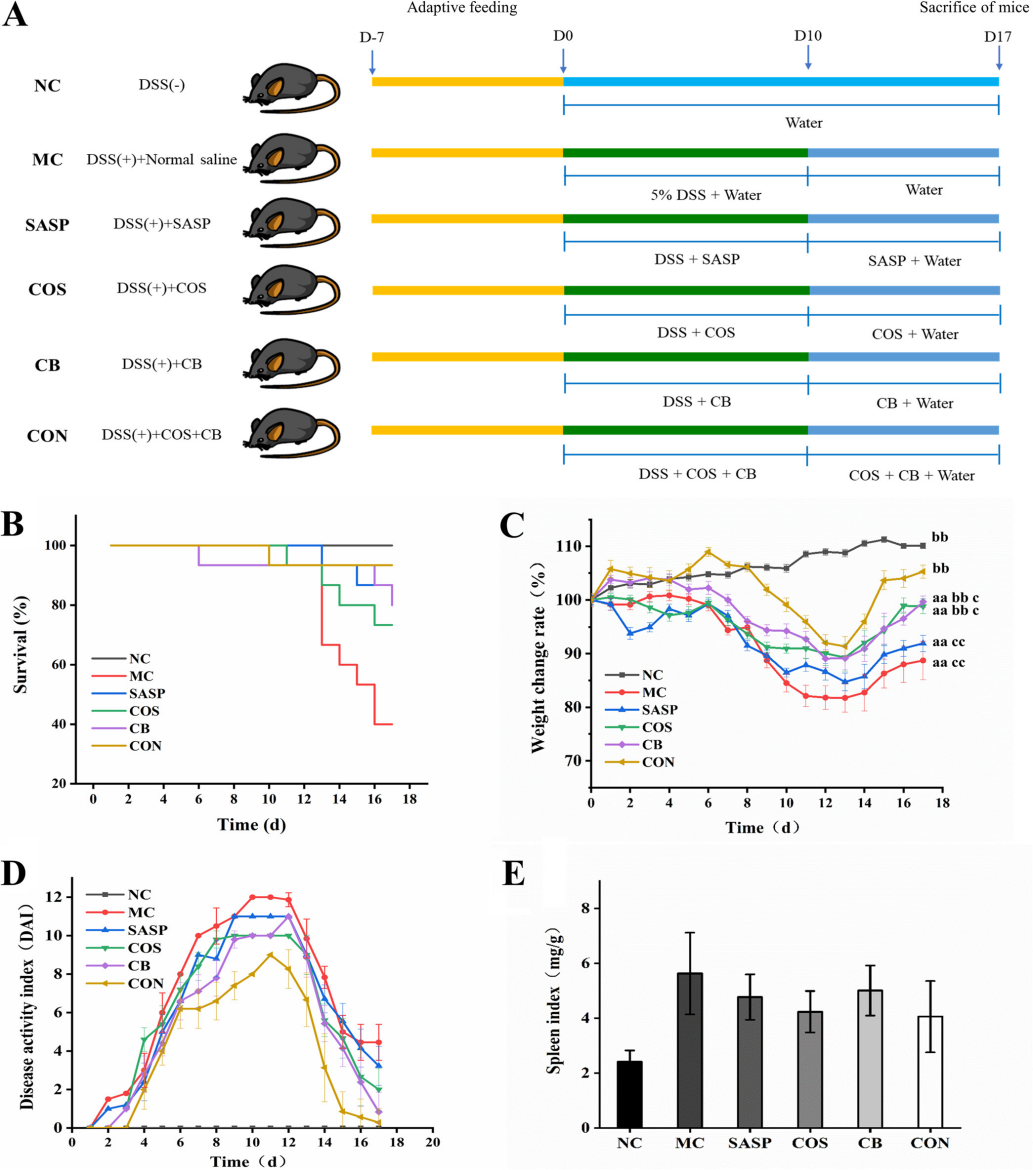
Effects of the C. butyricum and COS combination on survival, body weight, diet, DAI, and immune organ (spleen) index in DSS-induced UC mouse model. (A) Animal experiment design (schematic). (B) Survival rate during 17-day experimental period. (C) Weight change rate during 17-day period. (D) DAI score during 17-day period. (E) Spleen index on day 17. NC, normal group. MC, DSS-induced model group. SASP, SASP (500 mg/kg)-treated positive control group. COS, COS (MW, 2,500 Da; 200 mg/kg)-treated group. CB, C. butyricum (1 × 10^8^ CFU/mL)-treated group. CON, C. butyricum and COS combination-treated group. Data shown are mean ± SEM. a, *P* < 0.05; aa, *P* < 0.01 for comparison with NC. b, *P* < 0.05; bb, *P* < 0.01 for comparison with MC. c, *P* < 0.05; cc, *P* < 0.01 for comparison of SASP, COS, and C. butyricum with CON.

UC disease severity in mice is usually evaluated on the basis of mortality and body weight change rates. In our study, survival rates were high during the DSS induction period (days 1 to 10). Mortality rates began increasing gradually around day 13 in the DSS-induced model (MC), salazosulfapyridine (SASP), COS, and C. butyricum groups (Fig. 1B). At the end of the study period (day 17), survival rates for SASP, COS, C. butyricum, and C. butyricum and COS treatment (CON) were, respectively, 90%, 80%, 75%, and 95% and significantly (*P* < 0.01) lower for MC (55%). Mortality was significantly reduced for CON (i.e., C. butyricum and COS combination), whose synbiotic activity caused no adverse effects. For all groups, body weight change rate during the study period declined initially and then increased. Decrease (as percentage) of mean body weight relative to normal control (NC) was greatest for MC, least for CON, and intermediate for SASP, COS, and C. butyricum (Fig. 1C).

Disease activity index (DAI) is commonly used for evaluation of UC development and progression. All six groups showed an increase of DAI up to a maximum at day 10 or 12, followed by decline (Fig. 1D); however, details of the curves differed. On day 17, scores for COS, C. butyricum, CON, and SASP were significantly (*P* < 0.05 or *P* < 0.01) lower than for MC; notably, CON score was very close to that of NC (zero).

Organ index, the ratio of weight of a particular organ to body weight, is a parameter commonly used in toxicology studies. We calculated spleen index for evaluation of C. butyricum and COS effect. Spleen indices for the experimental groups were significantly (*P* < 0.01) higher than for NC, indicating occurrence of spleen hypertrophy or hyperplasia in our model (Fig. 1E). Indices for COS, C. butyricum, and SASP were significantly (*P* < 0.05 or < 0.01) lower than for MC. Index for CON was lower than for C. butyricum or COS, suggesting reversal of splenomegaly by C. butyricum and COS combination. The C. butyricum and COS combination had a stronger ameliorative effect on symptoms in our model than C. butyricum or COS alone.

### C. butyricum ± COS reduced morphological changes and injuries of colon tissues.

**FIG 2:**
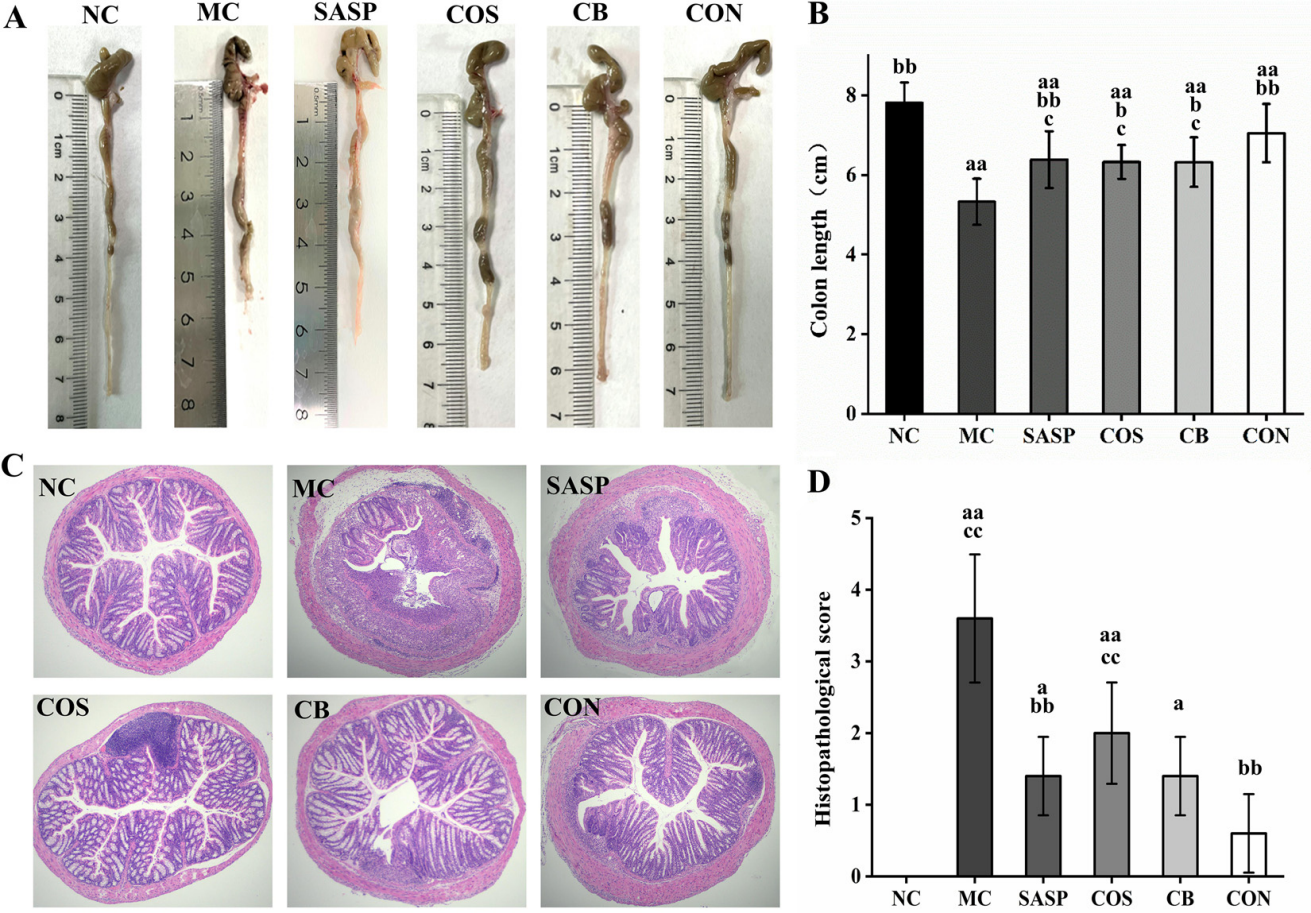
Effects of C. butyricum and COS on colon length and histopathological scores. (A) Representative colons from the six groups. (B) Colon length. (C) Histopathological (H&E) staining of colon tissue sections. (D) Histopathological scores. Notations as in Fig. 1.

Morphological changes of the colon, including shortened length and considerable tissue damage, generally occur in UC model mice. Colon length was significantly (*P* < 0.01) shorter for MC than for NC (Fig. 2A and B) and was effectively restored in COS and C. butyricum. Colon length was significantly (*P* < 0.05) greater for COS, C. butyricum, and CON than for MC, and CON was more effective (*P*< 0.05) than COS or C. butyricum alone in restoring length.

Histopathological scores of the groups, based on hematoxylin and eosin (H&E) staining of colon tissue sections, were compared. Colon tissue structure was severely damaged in the experimental groups but remained intact in NC (Fig. 2C). MC showed infiltration of numerous inflammatory cells and disappearance of large areas of goblet cells, glands, and crypts. In SASP, colon structure was mostly intact, but there was some damage from inflammatory cell infiltration and goblet cell disappearance. In C. butyricum and COS, colon structure was generally intact, aside from damage to a small portion and infiltration of a small number of inflammatory cells. In CON, colon structure was essentially normal, crypts were clearly visible, and goblet cells were arranged neatly.

Histopathological scores based on degree of colon tissue injury are shown in Fig. 2D. The score was significantly (*P* < 0.01) higher for MC than for NC, reflecting damage to intestinal structure by DSS treatment. Relative to MC, scores for SASP and CON were significantly (*P* < 0.01) lower, and those for C. butyricum and COS were slightly (not significantly) lower. These findings indicate that the C. butyricum and COS combination effectively ameliorated colon tissue injury in our UC model.

### C. butyricum ± COS ameliorated inflammation and oxidative stress levels.

**FIG 3:**
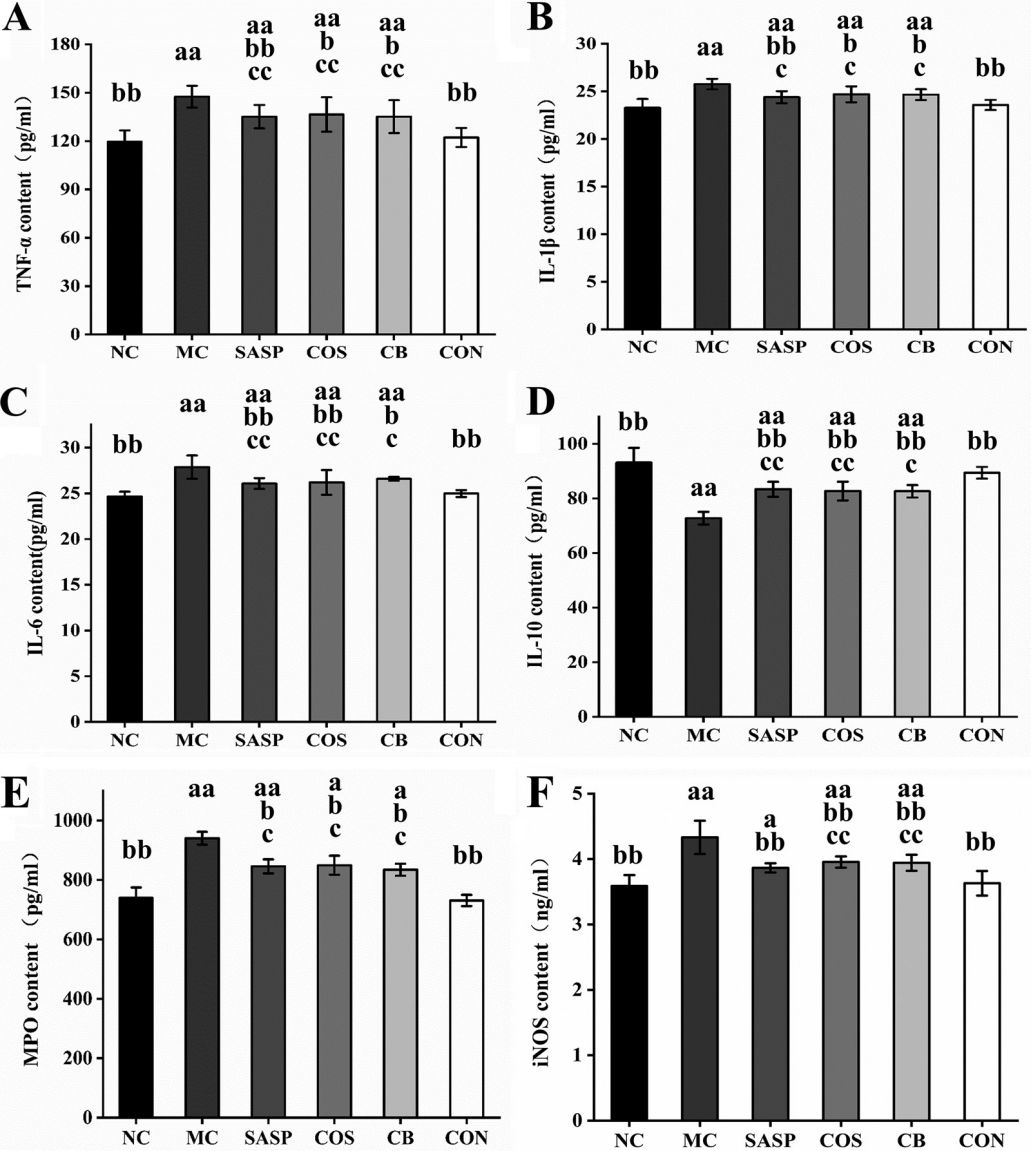
Effects of C. butyricum and COS on TNF-α (A), IL-1β (B), IL-6 (C), IL-10 (D), MPO (E), and iNOS (F) levels in colon tissues of the six groups. Notations as in Fig. 1.

We measured levels of proinflammatory and anti-inflammatory cytokines for evaluation of inflammation in our model. Relative to NC, levels for MC of three proinflammatory cytokines (TNF-α, IL-1β, IL-6) and of anti-inflammatory cytokine IL-10 were significantly (*P* < 0.01) higher and lower, respectively (Fig. 3), indicating that DSS induction caused severe inflammation. Relative to MC, levels of TNF-α, IL-1β, and IL-6 for COS, C. butyricum, and SASP were significantly (*P* < 0.05 or < 0.01) lower (Fig. 3A to C) and IL-10 levels for SASP, COS, C. butyricum, and CON were significantly (*P* < 0.01) higher (Fig. 3D). Levels of all of these cytokines for CON were close to those for NC. Thus, C. butyricum or COS alone relieved inflammation somewhat, but the C. butyricum and COS combination was more effective.

Myeloperoxidase (MPO) and inducible carbon monoxide synthase (iNOS) play important roles in development of inflammation. Levels of these two enzymes were significantly (*P* < 0.01) higher for MC than for NC and lower for SASP, COS, C. butyricum, and CON than for MC (Fig. 3E and F). MPO and iNOS levels for CON were close to those for NC, indicating a strong ameliorative effect of the C. butyricum and COS combination against inflammation and tissue damage in our model.

### C. butyricum ± COS regulated protein expression of TLR-4/NF-κB/MAPK signaling pathway.

**FIG 4:**
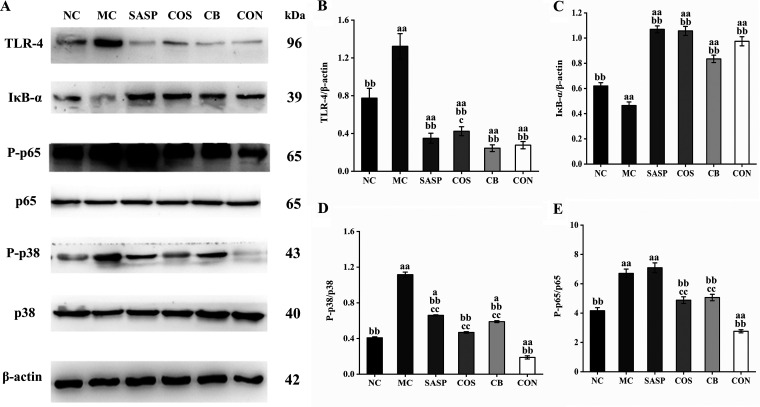
Expression levels of proteins involved in activation of NF-κB/MAPK signaling pathway. (A) Representative blots of TLR-4, IκB-α, P-p65, p65, P-p38, and p38 from Western blotting assay. (B to E) Bar graphs of TLR-4, IκB-α, P-p65/p65, and P-p38/p38 expression levels. Notations as in Fig. 1.

Expression of several proteins involved in the Toll-like receptor 4 (TLR-4)/NF-κB/mitogen-activated protein kinase (MAPK) signaling pathway was measured by Western blotting assay to clarify the mechanism of the anti-inflammatory effect of C. butyricum plus COS. Levels of TLR-4 (key receptor for lipopolysaccharide recognition by the innate immune system) were significantly (*P* < 0.01) higher for the experimental groups than for NC. TRL-4 expression was significantly (*P* < 0.01) downregulated for C. butyricum, COS, CON, and SASP (Fig. 4A and B). Level of IκB-α (repressor protein in the NF-κB pathway) was reduced for MC relative to that for NC but strongly (*P* < 0.01) upregulated for COS, C. butyricum, and SASP (Fig. 4A and C). Phosphorylation of p65 (protein involved in NF-κB pathway) and of p38 (involved in MAPK pathway) was higher for MC than for NC but was strongly reduced for COS, C. butyricum, and SASP. p-p65 and p-p38 levels were significantly (*P* < 0.01) lower for CON than for MC (Fig. 4A, D, and E).

### C. butyricum ± COS restored intestinal barrier function.

**FIG 5:**
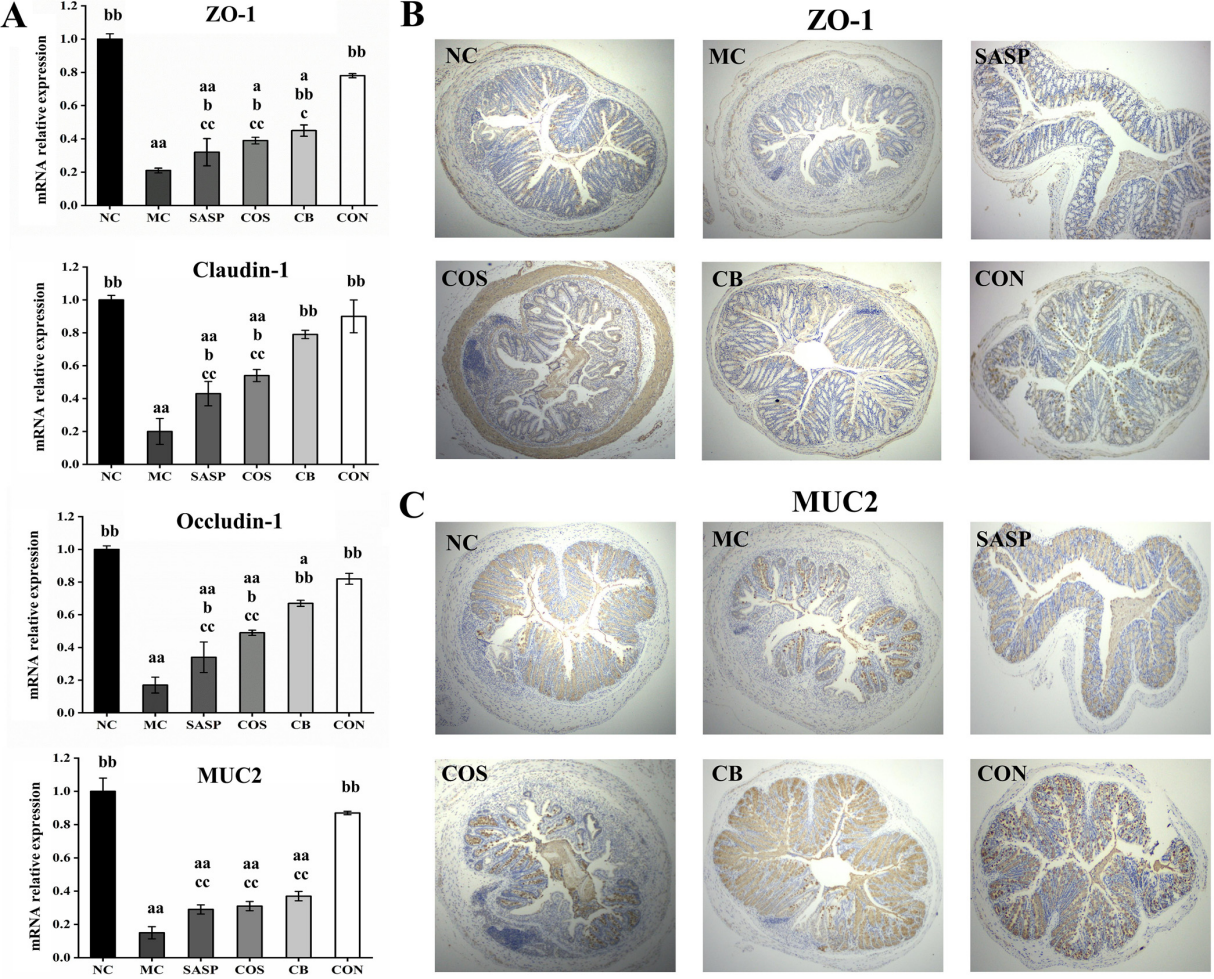
mRNA expression levels of ZO-1, claudin-1, occludin, and MUC2 (A) and immunohistochemical analyses of ZO-1 (B) and MUC2 (C) in colon. Notations as in Fig. 1.

Intestinal barrier function was evaluated based on expression levels of three tight junction (TJ) proteins (ZO-1, claudin-1, occludin) and of MUC2 glycoprotein in mucus. Reverse transcription quantitative PCR (RT-qPCR) analysis revealed that mRNA expression levels of these proteins were significantly (*P* < 0.01) lower for MC than for NC (Fig. 5A), consistently with features of the UC model. Transcriptional levels of the three TJ protein genes for COS, C. butyricum, and SASP were significantly (*P* < 0.05 or < 0.01) higher than for MC but much lower than in CON; in fact, levels for CON were close to those for NC. MUC2 expression levels for COS, C. butyricum, and SASP were higher than those for MC and did not differ significantly among themselves. MUC2 expression level was significantly (*P* < 0.01) higher for CON than for MC.

ZO-1 and MUC2 were subjected to immunohistochemical analysis. The ZO-1-positive area in epithelial cells (indicated by the brown color in figures) was much smaller for MC than for NC (Fig. 5B). The ZO-1-positive area was larger for COS, C. butyricum, and SASP and to a greater degree for CON. Findings from immunohistochemical analysis of MUC2 were similar (Fig. 5C). Thus, the C. butyricum and COS combination effectively restored intestinal barrier function in our model.

### C. butyricum ± COS modulated abundance and composition of gut microbiota.

**FIG 6:**
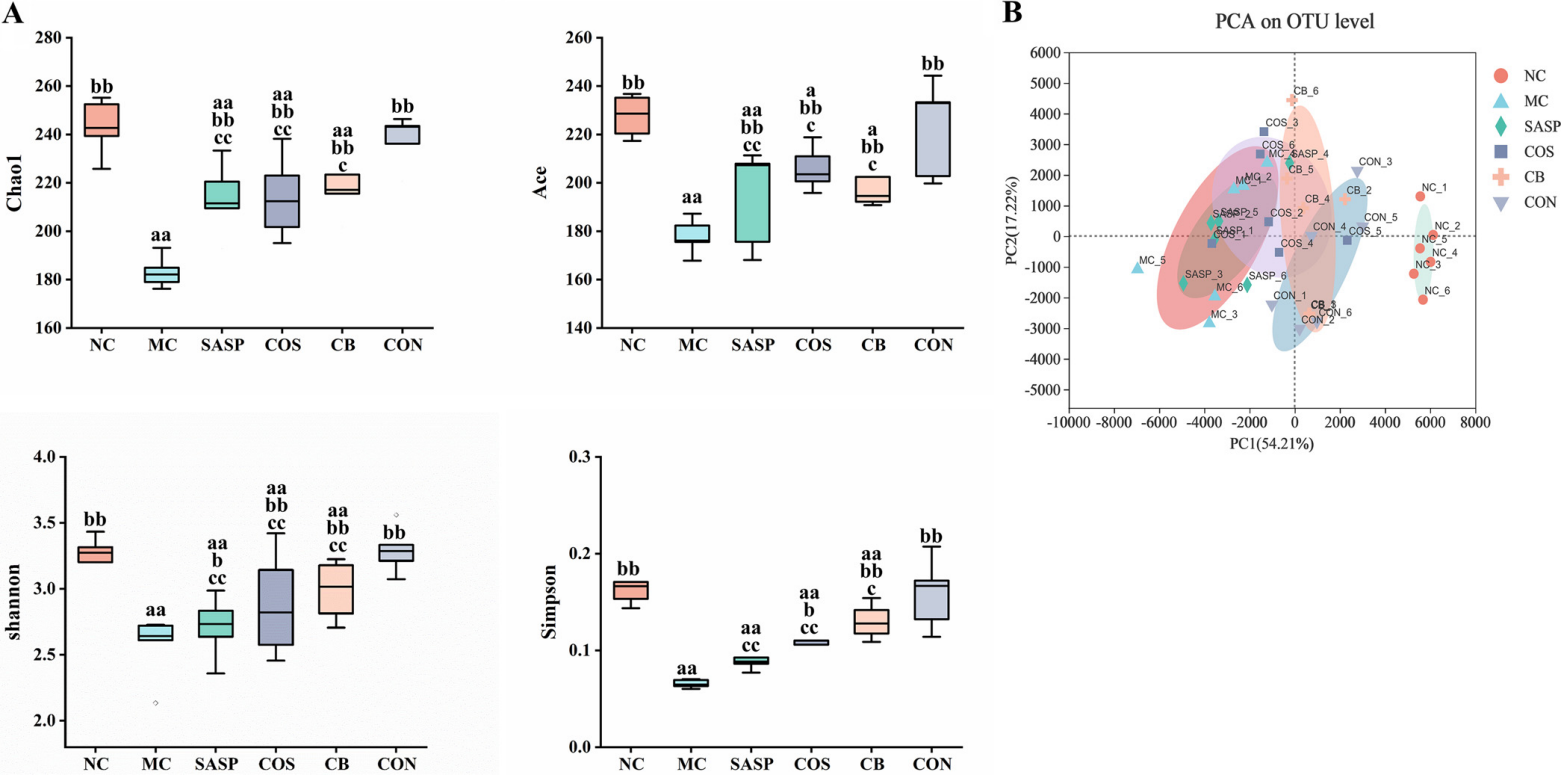
Alpha (A) and beta (B) diversity of fecal bacteria based on 16S rRNA gene sequencing. Notations as in Fig. 1.

Fecal samples were collected and subjected to 16S rDNA sequencing at the end of animal experiments. Changes of gut microbiota abundance were evaluated on the basis of α diversity, which reflects microbiota species richness and diversity. Chao1, Shannon, Ace, and Simpson indices were calculated based on operational taxonomic unit (OTU) numbers. Values of these four indices were significantly (*P* < 0.01) lower for MC than for NC but higher (*P* < 0.05 or < 0.01) for COS, C. butyricum, and SASP than for MC (Fig. 6A). Values of the indices for CON were close to those for NC. Principal-component analysis (PCA) revealed clear visual separation between MC and NC, indicating very different community compositions (Fig. 6B). Locations of SASP, COS, C. butyricum, and CON in the PCA graph were intermediate between MC and NC, with CON closest to NC. Thus, diversity and richness of gut microbiota were effectively restored by C. butyricum and/or COS (particularly in combination).

**FIG 7:**
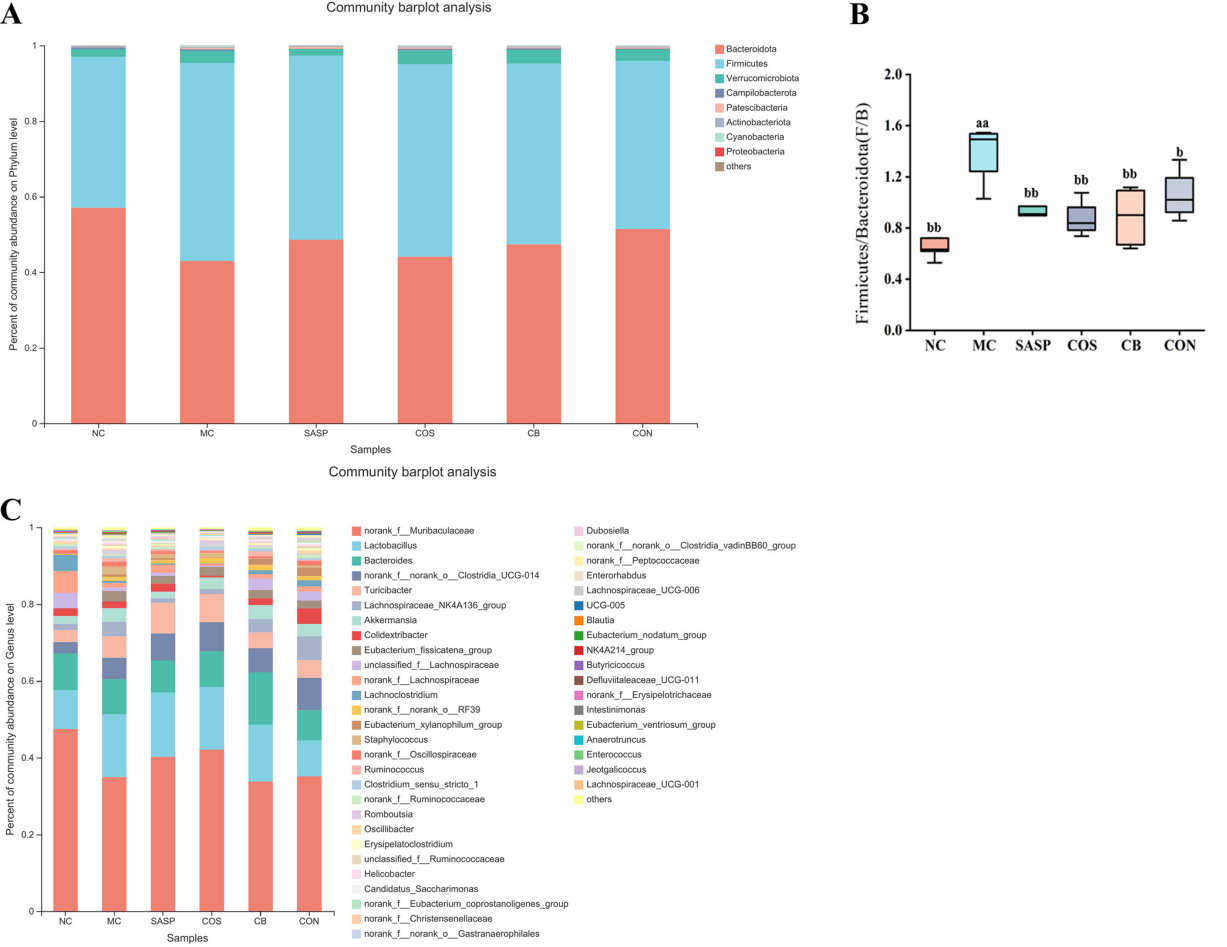
Effects of C. butyricum and COS on gut microbiota abundance in the six groups. (A) Phylum-level analysis. (B) Relative abundances of *Firmicutes*/*Bacteroidota* (termed F/B). (C) Genus-level analysis. (D) Relative abundances of *Muribaculaceae*, *Lactobacillus*, *Clostridia*_UCG-014, *Turicibacter*, *Ruminococcaceae*, *Butyricicoccus*, *Lachnospiraceae*_NK4A136, *Akkermansia*, *Enterorhabdus*, *Helicobacter*, *Bacteroides*, and *Erysipelatoclostridium*. Notations as in Fig. 1.

Analysis of gut microbiota changes at the phylum level indicated a predominance of *Bacteroidota*, *Firmicutes*, *Verrucomicrobiota*, *Campylobacterota*, *Patescibacteria*, *Actinobacteriota*, and *Proteobacteria*, but the relative abundance of these phyla differed among the experimental groups (Fig. 7A). The proportion of *Firmicutes* and *Bacteroidota* (termed F/B) was significantly (*P* < 0.01) higher for MC than for NC (Fig. 7B). F/B values for SASP, COS, C. butyricum, and CON groups were significantly (*P* < 0.05 or < 0.01) lower than that for MC. Analysis of gut microbiota changes at the genus level is presented as a community barplot in Fig. 7C and as relative abundances of selected genera in Fig. 7D. Relative to NC, the experimental groups showed significantly (*P* < 0.05 or < 0.01) lower abundances of beneficial genera (*Muribaculaceae*, *Lactobacillus*, *Clostridia*_UCG-014, *Turicibacter*, *Ruminococcaceae*, *Lachnospiraceae*_NK4A136, *Akkermansia*, and *Butyricicoccus*) and higher abundances of pathogenic genera (*Enterorhabdus*, *Erysipelatoclostridium*, *Bacteroides*, and *Helicobacter*) (Fig. 7D). Abundances of *Muribaculaceae*, *Lactobacillus*, *Clostridia*_UCG-014, *Turicibacter*, *Lachnospiraceae*_NK4A136, and *Butyricicoccus* were significantly (*P* < 0.05 or < 0.01) higher for COS, C. butyricum, and SASP than for MC and to an even greater degree for CON (Fig. 7D). Abundances of *Ruminococcaceae* and *Akkermansia* relative to MC were significantly (*P* < 0.01) higher for CON but not for COS or C. butyricum. Abundances of four pathogenic genera as above for CON were significantly (*P*< 0.01) lower than for MC, nonsignificantly lower than for COS, C. butyricum, and SASP groups, and essentially identical to NC (normal) values (Fig. 7D). These findings indicate a superior regulatory effect of the synbiotic C. butyricum and COS combination on gut microbiota composition and relative abundances of genera.

### C. butyricum ± COS increased intestinal content of short-chain fatty acids.

**FIG 8:**
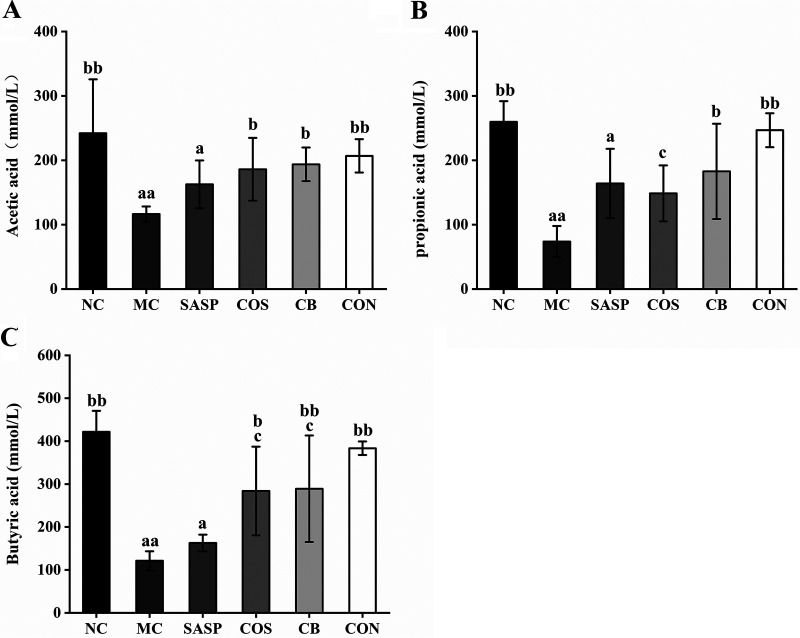
Effects of C. butyricum and COS on contents of SCFAs in the six groups. (A) Acetic acid. (B) Propionic acid. (C) Butyric acid. Notations as in Fig. 1.

Cecal contents of several SCFAs were analyzed. Contents of acetic acid, propionic acid, and butyric acid were significantly (*P* < 0.01) lower for MC than for NC (Fig. 8). Relative to MC, (i) acetic acid content was significantly higher for COS and C. butyricum (*P* < 0.05) and for CON (*P*< 0.01) (Fig. 8A), (ii) propionic acid content was significantly higher for C. butyricum (*P* < 0.05) and for CON (*P* < 0.01) (Fig. 8B), and (iii) butyric acid content was significantly higher for C. butyricum (*P* < 0.05), COS (*P* < 0.01), and to a greater degree for CON (Fig. 8C). None of the three SCFAs showed significantly higher content for SASP than for MC. These findings indicate that the C. butyricum and COS combination effectively raises SCFA levels, which are correlated with gut microbiota composition.

## DISCUSSION

C. butyricum and COS, applied singly as probiotic or prebiotic, have displayed excellent *in vitro* anti-inflammatory effects and *in vivo* anticolitis effects in previous studies. Addition of COS to culture medium did not inhibit C. butyricum proliferation or its production of butyric acid in our *in vitro* experiment (see Fig. S1 in the supplemental material). In a study of pigeon squabs, addition of COS and C. butyricum to diet enhanced growth and intestinal health (34). We hypothesized that synergistic (synbiotic) action of the C. butyricum and COS combination is more effective than either factor alone for prevention and/or therapy of UC. DSS treatment increases permeability of colonic epithelial cells, leading to passage of intestinal bacteria into the immune system and consequent intestinal inflammatory cell infiltration. DSS-induced UC is a widely used mouse model with symptoms (e.g., weight loss, diarrhea, and rectal bleeding) similar to those of human UC (35). We evaluated the synbiotic effects of C. butyricum plus COS in a DSS-induced UC mouse model.

C. butyricum plus COS treatment (CON group) effectively ameliorated UC symptoms as above in our *in vivo* model. DAI score and histopathological changes are commonly used indicators in assessment of UC severity (33). C. butyricum plus COS reversed DSS-induced increase of DAI score. Morphological observations showed that colon length was maintained and histopathological scores were reduced by C. butyricum plus COS, indicating the ability of this combination to ameliorate colonic inflammatory damage. This synbiotic effect was stronger than that of C. butyricum or COS alone.

UC is a chronic inflammatory disease of colonic mucosa, and the pathological process involves a variety of inflammatory cells that produce a variety of inflammatory cytokines. T helper cells produce anti-inflammatory cytokine (IL-10) as well as proinflammatory cytokines (TNF-α, IL-1β, IL-6) associated with intestinal barrier function that are driving forces in chronic inflammation and tissue damage (36, 37). TNF-α is involved in many immune and inflammatory responses and triggers intestinal inflammation by altering epithelial TJ structure and disrupting epithelial barrier function (38). IL-1β induces various proinflammatory mediators (cytokines, chemokines), resulting in extensive inflammatory responses. Colon tissues of UC mice and human patients expressed high levels of IL-1β mRNA (39). IL-1β and TNF-α stimulate lymphocytes to produce IL-6. Blocking of IL-6 or its receptors with monoclonal antibodies effectively inhibited progression of Crohn's disease (40, 41).

TNF-α, IL-1β, and IL-6 levels in the present study were significantly (*P* < 0.01) higher for MC than for NC, indicating progression of inflammation in our model. Levels of these proinflammatory cytokines were more strongly reduced for CON than for COS (*P* < 0.05) or C. butyricum (*P* < 0.01). In contrast, the level of anti-inflammatory factor IL-10 was significantly upregulated for CON. Hayashi et al. demonstrated that C. butyricum prevented experimental colitis in a mouse model by promoting IL-10 production by intestinal macrophages in inflamed mucosa and that IL-10-deficient macrophages did not display such preventive effect (42). IL-10 is involved in the etiology of IBD; gene mutation of IL-10 or its receptor results in spontaneous IBD development in mice and humans (43, 44). Anti-inflammatory effects of IL-10 include strong inhibition of synthesis of IL-6, TNF-α, and other proinflammatory factors at the transcriptional level (45). In our model, inflammation was significantly suppressed for C. butyricum and COS, and to a greater degree for CON, through upregulation of IL-10 and downregulation of TNF-α, IL-1β, and IL-6.

In the DSS-induced UC mouse model, the intestinal mucosal barrier is disrupted and microbes in the intestinal lumen enter the immune system, triggering a series of inflammatory reactions. Stimulation of Toll-like receptor 4 (TLR4) by its ligands results in signal transmission to the gene encoding region, and sequential activation of Iκ-B kinase (IKK) complex of NF-κB inhibitor, mitogen-activated protein kinase (MAPK), and NF-κB, with consequent activation of proinflammatory cytokines (e.g., IL-1, IL-6) and increased inflammation (46). TLR4 level is low in normal intestinal mucosa but upregulated during intestinal inflammation (47). In this study, DSS induction caused inflammation and significantly enhanced TLR4 level (MC versus NC). TLR4 level was significantly lower for CON, indicating an anti-inflammatory effect of the C. butyricum and COS combination in our model, consistently with findings of Tian et al. (48).

NF-κB, consisting of a heterodimer of RelA (p65) and p50, plays important roles in inflammatory processes and immune responses. Inhibitory protein IκB binds to NF-κB to form a trimeric complex, resulting in inactivation. IKK, upon stimulation by certain factors, phosphorylates the serine residue of the IκB subunit in the trimeric complex, leading to degradation of the IκB subunit and release of the NF-κB dimer (49). Activation of NF-κB signaling pathway induces secretion of TNF-α and IL-1β and exacerbates inflammation (50). COS was reported to reduce intestinal inflammation by inhibiting the NF-κB signaling pathway (33, 51). In a study of *Salmonella*-infected chickens, C. butyricum downregulated levels of proinflammatory cytokines (IFN-γ, IL-1β, IL-8, TNF-α) and reduced inflammatory reactions in intestinal epithelial cells by inhibiting the NF-κB signaling pathway (52). We observed a significant reduction of p65 level and an increase of IκB-α level for CON, indicating strong inhibition of the NF-κB signaling pathway. Such inhibitory effect was presumably associated with observed reduction of proinflammatory cytokine (TNF-α, IL-1β, IL-6) levels. We also examined expression levels of p38, which is involved in the MAPK pathway and in UC progression (53, 54). In IBD patients, p38 phosphorylation promoted recruitment and activation of lymphocytes and neutrophils and enhanced migration of circulating monocytes into inflammatory bowel tissues and their transformation into inflammatory macrophages, leading to exacerbation of bowel inflammation (55). p38 activation increased secretion of TNF-α and IL-1β, which further activated p38 (feedback regulation); this process also exacerbated inflammation. In the present study, the C. butyricum and COS combination, by suppressing activation of the p38/MAPK pathway, prevented such an inflammatory cytokine “storm.”

Myeloperoxidase (MPO) (an enzyme found in neutrophils) and inducible carbon monoxide synthase (iNOS) are commonly used as indicators for monitoring UC progression. Neutrophil activation leads to release of MPO into phagosomes, enhanced secretion of reactive oxygen, and acceleration of local intestinal inflammation (56). NO, an important inflammatory mediator and immune molecule, has strong *in vivo* biological activity and plays essential roles in tissue damage and inflammatory responses. NO level is correlated with UC severity. NF-κB activation leads to increased iNOS expression and NO release, with consequent tissue damage and inflammation (57). We observed increased MPO and iNOS levels in our UC mouse model and significant reduction of these levels in our CON group, indicating that the synbiotic C. butyricum and COS combination ameliorated oxidative stress level and inflammatory injury in colon tissue.

TJ proteins maintain intestinal mucosal cell barrier function, regulate intercellular exchange of beneficial substances, and prevent harmful substances from infiltrating the submucosa (58). Chelakkot et al. observed that impairment in UC patients of intestinal barrier integrity was associated with altered expression of TJ proteins (59). Claudin and occludin, transmembrane proteins characteristically expressed in certain tissues and cells, play key roles in maintenance of TJ barrier structure and function. In studies of filter-grown Caco-2 monolayers *in vitro* and mouse intestinal epithelial cells *in vivo*, Al-Sadi et al. demonstrated that occludin depletion by small interfering RNA (siRNA) knockdown resulted in selective increase in macromolecular flux (60). The cytosolic protein ZO-1 links transmembrane proteins to actin cytoskeleton and seals the epithelium, thus preventing epithelial fault localization that causes IBD (61). We observed significant downregulation of occludin, claudin-1, and ZO-1 expression levels in our mouse model, consistently with previous reports (33, 62). MUC2, the major component of mucus released by intestinal goblet cells, is essential for intestinal mucosal barrier formation (63). In a mouse UC model like ours, destruction of goblet cells caused reduced MUC2 expression and increased gut permeability to bacteria (64). In an *in vitro* study of human HT-29 colon cancer cells, Wang et al. observed that COS ameliorated DSS-induced mucus defects and upregulated MUC2 expression (65). Qi et al. reported that C. butyricum adhered to mucopolysaccharide sites on the HT-29 cell surface and promoted MUC2 production and glycosylation (66). In the present study, C. butyricum plus COS more strongly promoted expression of MUC2 and TJ proteins *in vivo* than did C. butyricum or COS alone, indicating a strong potential of the synbiotic combination for maintenance of intestinal barrier integrity and function.

Pathogenesis of chronic inflammatory diseases such as IBD and UC has been correlated with dysbiosis of the gut microbiome in previous studies. Relative to controls, UC patients displayed alteration of gut microbiota composition and significantly lower microbial diversity (67). In healthy subjects, bacterial phyla present in the intestinal tract are primarily *Bacteroidota* and *Firmicutes*, and smaller proportions of *Proteobacteria* and actinomycetes, whereas proportions of these and other groups in patients with inflammatory diseases are very different (68). In the present study, α diversity and PCA analyses revealed altered composition and reduced diversity of gut microbiota in our model. Amelioration of UC symptoms evidently depends on restoring balance and increasing diversity of gut microbiota through administration of probiotics, prebiotics, and/or synbiotics. Single administration of COS or C. butyricum was previously reported to be effective in regulation of gut microbiota composition and amelioration of colitis. COS treatment significantly increased gut microbiota α diversity and reversed phylum-, family-, or genus-level abundance changes of various intestinal bacteria (69). Our previous study showed that COS treatment reduced abundance of pathogenic bacteria (Escherichia coli/*Shigella*, *Proteobacteria*) and increased abundance of beneficial bacteria (*Ruminococcaceae*_UCG_014, *Prevotellaceae*_UCG_001) in our UC mouse model (33). C. butyricum regulates gut microbiota composition by enhancing probiotics (e.g., butyrate-producing bacteria, including *Lactobacillus*, *Ruminococcaceae*, and *Eubacterium*) and inhibiting pathogenic bacteria (7, 11, 70, 71). In hen cecum, C. butyricum treatment reduced E. coli levels but increased *Bifidobacterium* levels (52). In the present study, the C. butyricum and COS combination enhanced gut microbiota diversity in our model, and its effect was stronger than that of either component alone. Phylum-level abundance changes varied among our experimental groups. Proportion of F/B (see “*C. butyricum* ± COS modulated abundance and composition of gut microbiota”), an index of intestinal inflammation (72), was significantly (*P* < 0.01) higher for MC than for NC and lower for CON. Thus, the synbiotic C. butyricum and COS combination effectively promoted gut microbiota abundance and diversity in our model.

Our genus-level analysis indicated significantly higher levels of pathogenic bacteria (*Bacteroides*, *Erysipelatoclostridium*, *Enterococcus*, *Helicobacter*) for the experimental groups than for NC. Previous studies of IBD patients and mouse models reveal correlation of *Bacteroides* spp. abundance with disease severity, suggesting that these bacteria promote inflammation (73, 74). Certain *Bacteroides* strains were associated with colon cancer development, based on enterotoxin production (75). *Erysipelatoclostridium* is an opportunistic pathogen sometimes associated with metabolic syndrome, gout, or other diseases (76). We observed significantly higher *Erysipelatoclostridium* levels for our experimental groups than for NC, suggesting association of this genus with UC progression. *Enterococcus* and *Helicobacter* levels were also significantly higher for the experimental groups than for NC. A survey of mucosa-associated microbiota in IBD patients by Nishino et al. showed higher *Enterococcus* levels for Crohn's disease patients than for healthy control subjects (77). *Helicobacter* is a major pathogen and risk factor for chronic gastritis, peptic ulcer, gastric mucosa-associated lymphoid tissue lymphoma, and even gastric cancer. *Helicobacter* levels were elevated in UC model mice (78). In the present study, the synbiotic C. butyricum and COS combination had the strongest inhibitory effect on abundance of the above pathogenic bacterial genera.

Abundance of beneficial bacteria (*Muribaculaceae*, *Lactobacillus*, *Clostridia*_UCG-014, *Turicibacter*, *Lachnospiraceae*_NK4A136, *Akkermansia*, *Ruminococcaceae*, *Butyricicoccus*) is low in the gut of UC model mice, and is significantly enhanced by C. butyricum and/or COS treatment. Many intestinal pathogens depend on mucosal sugars as nutrients; therefore, commensal bacteria that compete with pathogens for such nutrients are “ecological gatekeepers” for maintenance of healthy homeostasis (79). Members of the family *Muribaculaceae* are able to metabolize mucin glycan (80), and may therefore inhibit proliferation of pathogenic bacteria in the gut by competing for mucin glycan sites. *Akkermansia*, a common mucin-degrading genus, promotes expression of transcriptional factor Foxp3-positive regulatory T (Treg) cells and IL-10 and suppresses inflammatory response (81). The C. butyricum and COS combination significantly elevated *Muribaculaceae* and *Akkermansia* levels in our model, suggesting that their synbiotic effect promotes intestinal tract stability and inhibits inflammation. C. butyricum plus COS also enhanced proliferation of *Lactobacillus*, *Clostridia*_UCG-014, *Turicibacter*, *Lachnospiraceae*_NK4A136, *Ruminococcaceae*, and *Butyricicoccus*, leading to increased production of SCFAs, which play important roles as an energy source for intestinal epithelial cells and in maintenance of colonic homeostasis (82). Disruption of gut microbiota balance leads to alteration of associated functions such as SCFA production.

**FIG 9:**
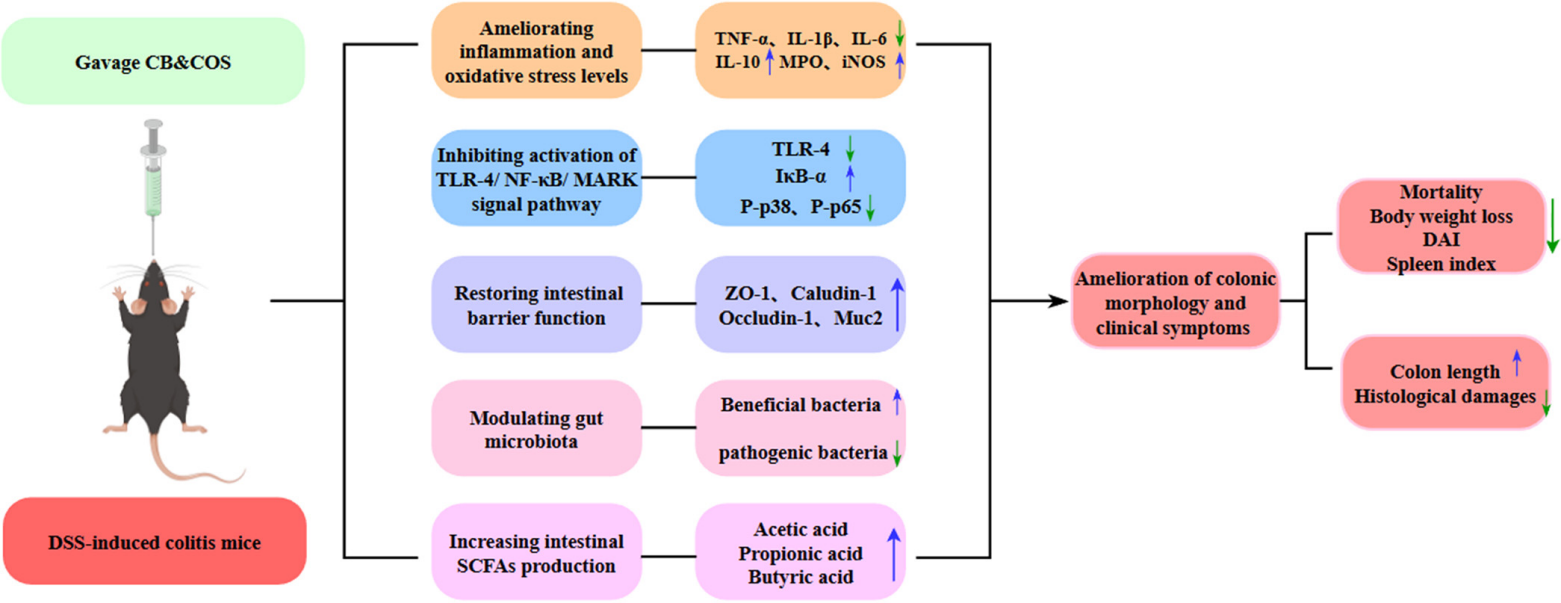
Proposed mechanism (schematic) for ameliorative effect of synbiotic C. butyricum and COS combination in our UC mouse model.

SCFAs have beneficial effects on intestinal mucosa, activate anti-inflammatory signaling cascades, and are involved directly or indirectly in cell proliferation, differentiation, and gene expression (83). SCFAs generally regulate host metabolism through two mechanisms. One is to regulate the expression of related genes by inhibiting histone deacetylase (HDAC). The other is by binding G-protein-coupled receptors (GPRs), in which FFAR2, FFAR3, and GPR109A are the main receptors activated by SCFAs, and they are expressed in various immune cells (eosinophils, basophils, neutrophils, and dendritic cells, etc.). SCFAs bind FFAR2 to regulate the change of flora and promote the production of immunoglobulin, bind FFAR3 to promote the differentiation of immune cells, and bind GPR109A to activate immune cells to regulate the secretion of cytokines (84–87). SCFAs reduced epithelial cell permeability and enhanced TJ protein activity and transmembrane resistance *in vitro* by regulating transcription of IL-10 receptors, occludin, ZO-1, and claudin (88, 89). They also increased thickness of mucosal layer in the human colon by upregulating MUC2 production (82). Gut dysbiosis was associated with reduced SCFA production in clinical trials and animal models (83). Likewise, fecal samples from IBD patients often show reduced SCFA levels (90). We measured contents of acetic acid, propionic acid, and butyric acid, the most abundant intestinal SCFAs. Relative to NC, SCFA contents were significantly lower for MC and higher for CON, reflecting differential alteration of gut microbial abundance. Zhang et al. similarly reported that COS treatment reversed abundance changes of various intestinal bacteria, thus promoting balanced production of intestinal metabolites (bile acids, tryptophan catabolites, SCFAs) (69). In this study, the C. butyricum and COS combination increased levels of beneficial bacteria (particularly SCFA producers) and suppressed levels of pathogenic bacteria, thus ameliorating UC symptoms through enhanced diversity and abundance of gut microbiota. A proposed mechanism (schematic) for this ameliorative effect is shown in Fig. 9. Our results demonstrate that C. butyricum plus COS regulates the disturbed gut microbiota, thereby increasing the beneficial metabolite of the gut microbiota, SCFAs, resulting in those beneficial effects on the host. Further study will be considered to reveal whether C. butyricum and COS directly affect the host or through intestinal flora by using fecal bacteria transplantation or germfree mice.

In conclusion, we used a DSS-induced UC mouse model to evaluate ameliorative effects of a synbiotic C. butyricum and COS combination. Findings clearly demonstrate that C. butyricum plus COS reduced clinical symptoms, improved colonic morphology, regulated inflammation-related cytokine levels, inhibited TLR-4/NF-κB/MAPK signaling pathway activation, maintained intestinal barrier function through enhanced expression of associated proteins, and promoted intestinal homeostasis by modulating gut microbiota composition and diversity. Effects of the C. butyricum and COS combination were stronger than those of either component alone. We conclude that the synbiotic C. butyricum and COS combination has strong potential as a therapeutic adjuvant for IBD/UC.

## MATERIALS AND METHODS

### Materials and reagents.

COS (average MW, 2,500 Da; degree of deacetylation [DDA], 91.3%; food grade) was prepared by enzymatic hydrolysis/membrane coupling method in a strictly endotoxin-free environment. Clostridium butyricum B1 CGMCC no. 4845 was cultured in reinforced clostridial medium (RCM) under anaerobic condition for 24 h at 37°C. DSS was from Shanghai Yuanye Biotech Co. Salazosulfapyridine (SASP) was from TCI Chemicals Co. (Shanghai). Myeloperoxidase (MPO), inducible nitric oxide synthase (iNOS), TNF-α, IL-1β, IL-6, and IL-10 enzyme-linked immunosorbent assay (ELISA) kits were from Jiangsu Meimian Industrial Co. RT-qPCR and fluorescence-based quantitative PCR kits were from Nanjing Vazyme Biotech Co. Primers for qPCR were synthesized by Tsingke Biotech Co. (Wuhan, China). Antibodies directed to β-actin, Toll-like receptor 4 (TLR4), IκB-α, NF-κB p65, phosphor(p)-NF-κB p65, p38, and phosphor(p)-p38 were from Cell Signaling Technology (Beverly, MA, USA). Enhanced chemiluminescence (ECL) system was from Shanghai Tanon Co. Bicinchoninic acid (BCA) protein assay kit, protein phosphatase inhibitor mixtures (catalog no. P1045), radioimmunoprecipitation assay (RIPA) protein lysis buffer (catalog no. P0013B), SDS-PAGE gel preparation kit, tissue fixation solution/4% paraformaldehyde, dried skim milk, horseradish peroxidase (HRP)-labeled goat anti-rabbit IgG, and RNeasy animal RNA extraction kit were from Beyotime Institute of Biotechnology (Shanghai). Other reagents were from Sinopharm Chemical Reagent Co. (Shanghai).

### Animal experiments.

Our UC mouse model was as described previously (33) with minor modification. Experimental procedures were approved by the Animal Care and Use Committee of Huazhong Agricultural University (certificate no. SYXK2016-0057) and performed in accordance with internationally accepted guidelines and ethical principles. C57BL/6 mice (male; 6 to 8 weeks old) were housed at the Laboratory Animal Research Center of Huazhong Agricultural University, maintained at 25°C under 12 h light/12 h dark cycle with *ad lib* access to standard lab food pellets and water and allowed to adapt to the environment for 7 days prior to experiments. Animal experiment design is shown schematically in Fig. 1A. Mice (total 120) were assigned randomly to 6 groups, each with *n* = 20. Group I was the normal control, administered intragastrically with 0.9% normal saline solution for 17 days (termed “NC”). Group II was the UC mouse model control, induced by 5% (wt/vol) DSS for 10 days and then administered with normal saline as above (termed “MC”). Groups III to XI were DSS-induced for 10 days and then administered for 17 days with SASP (500 mg/kg; drug often used in UC treatment; positive control), COS (200 mg/kg), C. butyricum (1 × 10^8^ CFU/mL), or the C. butyricum and COS combination (respectively termed “SASP,” “COS,” “C. butyricum,” and “CON”). Body weight and food intake were recorded and fecal samples collected every day during the 17-day period. Survival rate in each group was calculated as the following formula: Survival rate (%) = number of living mice / total number of mice in each group × 100.

At the end of the study period, mice were sacrificed (cervical dislocation), subjected to abdominal disinfection, and dissected.

### Colonic morphology and histopathological scoring.

Colon tissues were collected, length measured, and morphological changes observed through hematoxylin and eosin (H&E) staining. For evaluation of intestinal inflammation, distal colon tissues were rinsed with 0.9% normal saline, dried with filter paper, fixed with 4% paraformaldehyde solution, washed under running water, dehydrated by ethanol, paraffin-embedded, cut into slices, deparaffinized, and H&E stained. Histopathological damage was scored using the following four categories based on inflammation severity, crypt disappearance, and pathological changes: 0, normal intestinal mucosa; 1, mild inflammation and edema in mucosal layer, disappearance of one-third of basal crypts; 2, moderate mucosal inflammation, disappearance of ⅔ of crypts; 3, moderate mucosal inflammation, complete disappearance of crypts, epithelium remains intact; 4, severe inflammation of mucosa, submucosa, and muscularis mucosa, disappearance of crypts and epithelium (91).

### Disease activity index scoring.

UC progression was evaluated by DAI scoring of clinical parameters as follows: body weight loss (0, <1%; 1, 1 to 5%; 2, 6 to 10%; 3, 11 to 15%; 4, >15%), stool consistency (0, normal; 1, soft but still formed; 2, very soft; 3, very soft and unformed; 4, loss of form/diarrhea); fecal bleeding (0, normal; 1 to 2, hemoccult positive; 3, visible bleeding in stool; 4, rectal bleeding) (33).

### Inflammatory cytokines and oxidative stress kinases.

Blood was collected from orbital venous plexus, centrifuged (2,500 × *g*, 10 min, 4°C), and supernatant collected. Serum levels of inflammatory cytokines (TNF-α, IL-6, IL-1β, IL-10) and oxidative stress kinases (MPO, iNOS) were assayed using ELISA kits as per manufacturer’s protocols.

### Reverse transcription quantitative PCR.

mRNA expression levels of occludin, claudin-1, ZO-1, and MUC2 were determined by RT-qPCR. Total RNA was extracted from RNAeasy animal RNA isolation kit with spin column, and reverse transcribed into cDNA using HiScript II Q RT SuperMix for qPCR (+gDNA wiper) as per manufacturer’s protocols. Sequences of primers used are listed in Table S1 in the supplemental material. β-actin was the internal reference. RT-qPCR was performed using AceQ qPCR SYBR green master mix with the following program: 95°C for 3 min; 40 cycles of 95°C for 10 s, 60°C for 20 s, 95°C for 15 s; 60°C for 1 min; and 95°C for 15 s. Reactions were performed in triplicate. Fold changes of genes were calculated using the 2^−ΔΔ^*^CT^* method.

### Immunohistochemical analysis.

The method of Chen et al. (10), with minor modification, was used to conduct immunohistochemical analysis. Colon tissue sections were deparaffinized, rehydrated, treated with citrate buffer (pH 6.0) for antigen retrieval, washed with phosphate-buffered saline (PBS), incubated in 3% H_2_O_2_ to eliminate endogenous peroxidase activity, blocked with goat serum, incubated with anti-ZO-1 (catalog no. GB111402; 1:500 dilution; Servicebio) or anti-MUC2 antibody (catalog no. GB14110; 1:500 dilution; Servicebio) overnight at 4°C, washed with PBS, covered with HRP-labeled secondary antibody, incubated at room temperature for 50 min, visualized by 3,3′-diaminobenzidine (DAB) staining, counterstained with hematoxylin, and evaluated by light microscopy.

### Western blotting assay.

Colon tissues (30 mg) were washed with PBS, homogenized, and centrifuged (12,000 × *g*, 15 min, 4°C). Total protein content of the supernatant was measured using a BCA kit, and expression levels of TLR4, IκB-α, p65, p38, p-p65, and p-p38 were determined by Western blotting assay. Protein samples (equal amounts) were separated by SDS-PAGE and electrotransferred onto polyvinylidene difluoride (PVDF) membranes. Membranes were blocked with 5% powdered nonfat milk for 2 h at room temperature, incubated with primary antibodies overnight at 4°C, incubated with corresponding HRP-labeled secondary antibodies for 2 h at room temperature, and visualized using an ECL system (92).

### Fecal microbiome sequencing and analysis.

Total genomic DNA was extracted from fecal samples using QIAamp DNA stool minikit (Qiagen, Hilden, Germany) as per manufacturer’s protocol. PCR amplification of bacterial 16s rRNA V3-V4 hypervariable regions was performed using primers 343F (5′-TACGGRAGGCAGCAG-3′)/798R (5′-AGGGTATCTAATCCT-3′). PCR amplicons were sequenced using the Illumina MiSeq platform (Illumina, San Diego, CA, USA) as per standard protocols of Shanghai Majorbio Bio-Pharm Technology Co. Paired-end reads were merged, demultiplexed, and subjected to quality control implementation using software programs Trimmomatic and FLASH. Resulting optimized sequences were clustered into operational taxonomic units (OTUs) having >97% similarity by multithreaded 64-bit tool VSEARCH. Gut microbiota composition was analyzed using QIIME-based microbiome bioinformatics platform as described previously (33, 93).

### SCFA analysis.

Cecum content samples were mixed with acidified pure water, homogenized, and centrifuged, with 2-ethylbutyric acid as internal standard. Supernatants were filtered through microfiltration membrane (pore size, 0.22 μm), collected in sample bottles, and stored at −20°C. SCFAs were analyzed by gas chromatography, using a GC2010 Plus system (Shimadzu, Kyoto, Japan) with a DB-FFAP column (Agilent Technologies, Santa Clara, CA, USA). Gas chromatography (GC) conditions were as follows: carrier gas, nitrogen; flow rate, 1.20 mL/min; injector temperature, 240°C; oven temperature, 200°C; sample injection volume, 1 μL.

### Statistical analysis.

Data were expressed as mean ± standard error of the mean (SEM). Statistical analysis was performed using software program SPSS for Windows v. 20.0 (SPSS Inc.; Chicago, IL, USA). Differences between means were considered to be significant for *P* < 0.05 and highly significant for *P* < 0.01.
