# Supplementary material for: Clostridium butyricum and Chitooligosaccharides in Synbiotic Combination Ameliorate Symptoms in a DSS-Induced Ulcerative Colitis Mouse Model by Modulating Gut Microbiota and Enhancing Intestinal Barrier Function
Source: Microbiol Spectr. 2023 Mar 28;11(2):e04370-22. doi: 10.1128/spectrum.04370-22 (PMC10100383; doi:10.1128/spectrum.04370-22)
Supplement: Supplemental file 1 — Supplemental material. Download spectrum.04370-22-s0001.pdf, PDF file, 0.2 MB [file spectrum.04370-22-s0001.pdf]

Supplemental Data

Fig. S1. Effect of COS on in vitro proliferation and acid production of CB. (A) Proliferation of CB on reinforced clostridial medium (RCM) with four carbon sources (a-d) as below. (B) Acetate content. (C) Butyrate content. a: no carbon source. b: COS (5 g/L) as sole carbon source. c: COS (2.5 g/L) + glucose (2.5 g/L) as complex carbon source. d: COS (5 g/L) + glucose (5 g/L) as complex carbon source. * p< 0.05; ** p< 0.01.

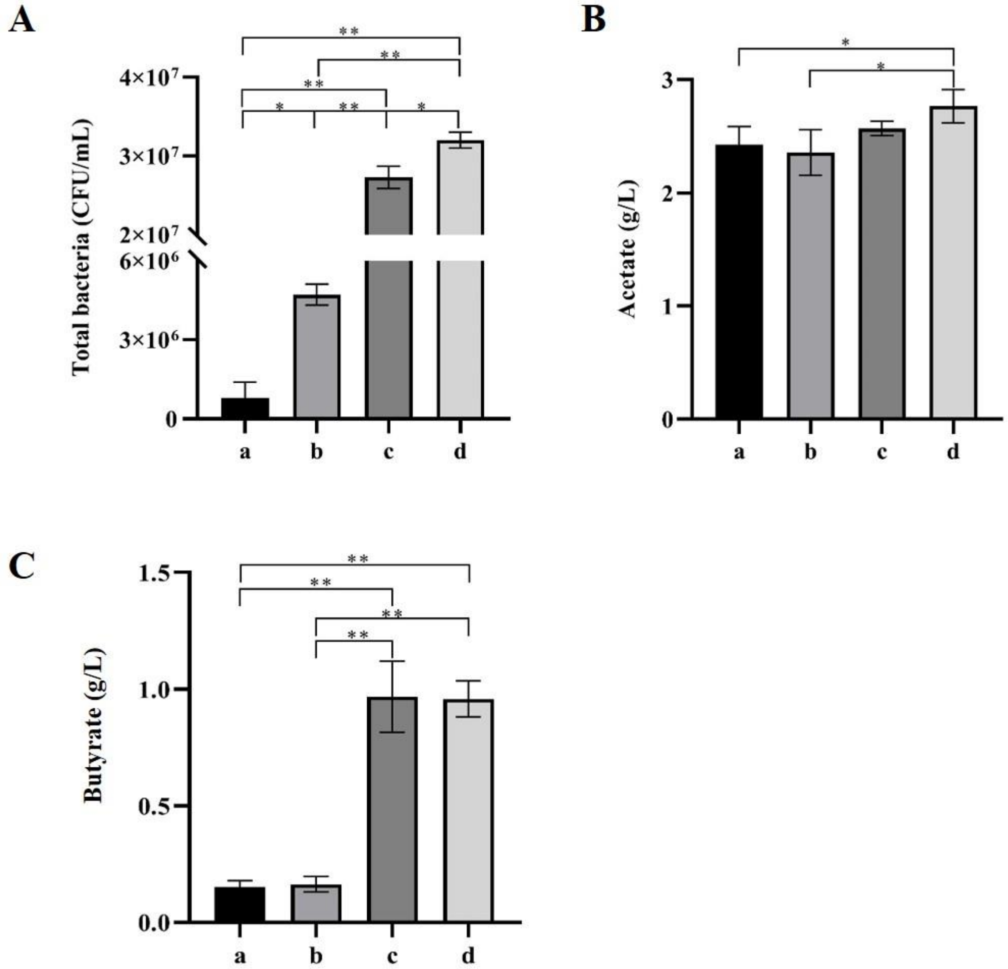

Table S1. Sequences of primers used for RT-qPCR.

**Target gene**	**Forward primer (5´-3´)**	**Reverse primer (5´-3´)**
β-actin	CTACCTCATGAAGATCCTGACC	CACAGCTTCTCTTTGATGTCAC
ZO-1	GCGAACAGAAGGAGCGAGAAGAG	GCTTTGCGGGCTGACTGGAG
occludin	TGGCTATGGAGGCGGCTATGG	AAGGAAGCGATGAAGCAGAAGGC
claudin-1	GCTGGGTTTCATCCTGGCTTCTC	CCTGAGCGGTCACGATGTTGTC
MUC2	ATGCCCACCTCCTCAAAGAC	GTAGTTTCCGTTGGAACAGTGAA
